# Mothers and Babies Virtual Group (MBVG) for perinatal Latina women: study protocol for a hybrid type-1 effectiveness-implementation randomized controlled trial

**DOI:** 10.1186/s13063-024-08423-z

**Published:** 2024-09-11

**Authors:** Rheanna Platt, Sarah Polk, Alinne Z. Barrera, Sandraluz Lara-Cinisomo, Lisa R. Hirschhorn, Andrea K. Graham, Rashelle J. Musci, Jaime Hamil, Diane Echavarria, Lindsay Cooper, S. Darius Tandon

**Affiliations:** 1 Department of Psychiatry and Behavioral Sciences & Division of Child and Adolescent Psychiatry, Johns Hopkins University/Johns Hopkins Bayview Medical Center, 5500 East Lombard St, Baltimore, MD 21224 USA; 2 Johns Hopkins University School of Medicine, Baltimore, MD 21224 USA; 3 Centro SOL, Johns Hopkins University School of Medicine, Baltimore, MD 21224 USA; 4 Palo Alto University, 1791 Arastradero Road, Palo Alto, CA 94304 USA; 5 University of Illinois Urbana-Champaign, Champaign, IL 61820 USA; 6 Northwestern University Feinberg School of Medicine, Chicago, IL 60611 USA; 7 Johns Hopkins Bloomberg School of Public Health, Baltimore, MD 21205 USA

**Keywords:** Postpartum depression, Prevention, Group intervention, Telehealth, Latina, Intervention

## Abstract

**Background:**

Immigrant Latinas (who are foreign-born but now reside in the USA) are at greater risk for developing postpartum depression than the general perinatal population, but many face barriers to treatment. To address these barriers, we adapted the Mothers and Babies Course—an evidence-based intervention for postpartum depression prevention—to a virtual group format. Additional adaptations are inclusion of tailored supplemental child health content and nutrition benefit assistance. We are partnering with Early Learning Centers (ELC) across the state of Maryland to deliver and test the adapted intervention.

**Methods:**

The design is a Hybrid Type I Effectiveness-Implementation Trial. A total of 300 participants will be individually randomized to immediate (*N* = 150) versus delayed (*N* = 150) receipt of the intervention, Mothers and Babies Virtual Group (MB-VG). The intervention will be delivered by trained Early Learning Center staff. The primary outcomes are depressive symptoms (measured via the Center for Epidemiologic Studies-Depression Scale), parenting self-efficacy (measured via the Parental Cognition and Conduct Towards the Infant Scale (PACOTIS) Parenting Self-Efficacy subscale), and parenting responsiveness (measured via the Maternal Infant Responsiveness Instrument) at 1-week, 3-month, and 6-month post-intervention. Depressive episodes (Structured Clinical Interview for DSM-V- Disorders Research Version) at 3-month and 6-month post-intervention will also be assessed. Secondary outcomes include social support, mood management, anxiety symptoms, perceived stress, food insecurity, and mental health stigma at 1-week, 3-month, and 6-month post-intervention. Exploratory child outcomes are dysregulation and school readiness at 6-month post-intervention. Intervention fidelity, feasibility, acceptability, and appropriateness will also be assessed guided by the Reach, Effectiveness, Adoption, Implementation, Maintenance (RE-AIM) framework.

**Discussion:**

This study will be one of the first to test the efficacy of a group-based virtual perinatal depression intervention with Latina immigrants, for whom stark disparities exist in access to health services. The hybrid effectiveness-implementation design will allow rigorous examination of barriers and facilitators to delivery of the intervention package (including supplemental components) which will provide important information on factors influencing intervention effectiveness and the scalability of intervention components in Early Learning Centers and other child-serving settings.

**Registration.:**

ClinicalTrials.gov NCT05873569.

**Supplementary Information:**

The online version contains supplementary material available at 10.1186/s13063-024-08423-z.

## Background

Between 10 and 22% of pregnant individuals in the USA develop postpartum depression (PPD) [1–4]. The effects of PPD extend across generations, with studies demonstrating that depressed mothers exhibit lower parenting self-efficacy [5, 6], less responsiveness to their child’s cues [7, 8] and less positive emotion [9] when engaging with their young children compared to non-depressed mothers. Immigrant Latinas (individuals who are foreign-born but now reside in the USA) represent a diverse population that has been shown to be at greater risk for developing PPD than the general perinatal population [10–13] due to a range of sociocultural and structural factors including acculturative stress, family separation, and high rates of trauma, poverty, and discrimination [10, 14]; perinatal is defined as pregnancy and the first year postpartum. However, despite elevated risk for PPD, immigrant Latinas have low rates of engagement with traditional mental health services due to a range of barriers (e.g., lack of insurance, competing demands, stigma) [15], suggesting the need to deliver interventions outside of traditional healthcare settings [16, 17].

Systematic reviews, including a 2019 United States Preventive Services Taskforce (USPSTF) review, highlight effective psychological interventions for preventing PPD onset and reducing depressive symptoms [18, 19]. The USPSTF recognized Mothers and Babies (MB), an intervention with content specific to the perinatal period built on cognitive-behavioral therapy (CBT) and attachment theory, as one of the two most efficacious interventions for preventing PPD. A series of randomized controlled trials (RCTs) demonstrate MB’s impact on reducing maternal depressive symptoms [20–23] and prevention of new cases of major depression [20, 21, 23]. MB was developed and tested via two RCTs with immigrant, Spanish-speaking Latinas [21, 23]. Low social support is a potent risk factor for PPD [24, 25], particularly salient among immigrant women [26, 27]. As such, several PPD preventive interventions—including MB—are delivered in a group format [18, 19]. Group preventive interventions targeting PPD [21, 23, 28] and other health conditions [29] have been well-received by immigrant Latinas [37, 38].

Despite evidence of effectiveness among immigrant Latinas, previous MB trials with Latinas have had several limitations, including variability in intervention dosage. In one trial [21], Latina participants averaged just over 50% attendance and greater intervention dosage was associated with increased effects on depressive symptoms; participants receiving > 50% of sessions had a − 0.53 effect size for reduction in depressive symptoms versus a − 0.28 effect size among all MB recipients.

EHealth interventions (delivered via telephone, apps, or web-based platforms) [30] mitigate several barriers to engaging perinatal immigrant Latinas in PPD interventions including transportation, childcare, and scarcity of language-concordant providers. Multiple reviews suggest that eHealth interventions demonstrate small to medium effect sizes in preventing or treating PPD [31, 32]. The COVID-19 pandemic required a transition from in-person to virtual interventions with early reports of success including among group interventions delivered virtually to immigrant adults with limited English proficiency [33]. There is evidence for the acceptability of a range of eHealth approaches and of increasing internet use among immigrant Latinos [29, 34, 35]. Virtual interventions can also address system-level barriers by promoting efficient use of bilingual staff [36, 37]. Previous studies have found high satisfaction and engagement with *virtua*l group interventions among immigrant Latinas for a range of conditions, including diabetes [38] and cancer [39, 40]. However, despite acceptability of virtual group interventions among Latinas [40] and the advantage of a virtual group modality to mitigate both risks for PPD and barriers to engagement among immigrant Latinas, few prior studies have examined the use of a virtual group-based preventive intervention for PPD with this population.

### MB adaptation for virtual group delivery

To address gaps in PPD preventive intervention delivery to immigrant Latinas, our team recently completed a pilot study in which we delivered MB virtually via Zoom: Mothers and Babies Virtual Group (MB-VG) [41]. MB-VG was developed in close collaboration alongside key community partners (e.g., service providers). While retaining key intervention components, we made four main modifications to the in-person MB group intervention. *First*, MB-VG’s delivery was changed from six sessions each lasting 90–120 min to 10 sessions each lasting ~ 60–75 min. Given the variability in dosage found in previous MB trials with immigrant Latinas, as well as practical barriers (e.g., childcare), partners believed shorter sessions would be more acceptable and feasible. *Second*, MB-VG added a “Resource Advocate” to facilitate participants’ connection to nutrition support, directly addressing food insecurity—a key social determinant of health associated with PPD among immigrant Latinas [42], and barriers to enrollment in nutrition assistance programs such as the Supplemental Nutrition Assistance Program (SNAP) [43]. Resource Advocates are rigorously trained Spanish/English bilingual undergraduate students supervised by medical school faculty and staff [44]. *Third,* due to partners’ desire for additional child health and development-focused guidance around topics including infant growth, development, and behavior, we incorporated a pediatric health care provider into sessions, with the provider joining virtual session for 15 min at the conclusion of the core MB content (see Table 1 for outline of content delivered by pediatric health care provider, for which a session-by-session manual was also developed). *Fourth*, MB-VG includes text messaging to promote session attendance, encourage skill practice, and reinforce health-related content delivered by the pediatric health care provider. We will send text messages 24–72 h apart via the HealthySMS system, a web-based platform used to deploy text messages in previous MB studies [45] and other studies of mental health interventions [46, 47].

**Table 1:** MB-VG content, by intervention session

MB m odule	MB session	Core MB content found in session (Each session reinforced with text messages)	Pediatrician topics
**Introductory**	**1**	Introduction to MB-VG	Introduction to Pediatrician & Resource Advocate and an overview of SNAP benefits
**Pleasant** **Activities**	**2**	Pleasant activities and my mood	Nutrition: Healthy Infant Feeding
	**3**	Pleasant activities and your baby	Development: Promoting Language Development
**Thoughts**	**4**	Thoughts and how they affect our mood	Development: Soothing a Crying Baby
	**5**	Identifying and modifying unhelpful thoughts	Nutrition: “Milk” choices
	**6**	Relationship between mood, thoughts, and future; promoting child’s healthy thinking	Nutrition: Health Infant Growth
**Contact** **With Others**	**7**	Contact with other people and my mood	Development: Teaching Boundaries
	**8**	Social support network for mother and child	Nutrition: Beverage Options
	**9**	Communication style and mood; role transitions with new child in home	Development: Promoting bilingualism
**Review**	**10**	Review and planning for the future	Wrap Up: Transition to Pediatrician

## Methods/design

### Study aims and hypotheses

Aim 1: To examine MB-VG effectiveness with perinatal immigrant Latinas at risk of PPD. Women receiving MB-VG will exhibit greater reductions in depressive symptoms (Hypothesis 1), exhibit fewer cases of PPD (H2) and report increased parenting self-efficacy and responsiveness (H3) compared to control participants receiving usual family support services. Exploratory Aim: Among enrolled participants (*n* ~ 150) who have an older child aged 2.5–4.5 years, we will examine whether the skills taught in MB-VG also promote less child dysregulation and greater child readiness for school. Aim 2. To evaluate MB-VG implementation. To inform future intervention delivery and scalability, we will assess key implementation outcomes. Guided by the Reach, Effectiveness, Adoption, Implementation, Maintenance (RE-AIM) framework [48], we will use mixed methods (e.g., semi-structured interviews, survey data, and session audio-recordings) to assess MB-VG reach, adoption, implementation, and maintenance. Aim 3. To examine contextual factors influencing MB-VG effectiveness and implementation. Guided by the Consolidated Framework for Implementation Research (CFIR) [49], we will measure contextual factors at the outer, inner, actors, intervention, and implementation process (virtual) levels via mixed methods. Publications will be developed for each study aim, with authorship determined in accordance with ICMJE guidelines. SPIRIT reporting guidelines are used in this manuscript [50].

### Setting: Maryland’s Judy Centers

Enacted in 2000 by the Maryland State Legislature, Judith P. Hoyer Early Learning Hubs (“Judy Centers”) are a network of center-based early childhood programs aimed at promoting kindergarten readiness of children from low-income families [51]. There are currently 85 Judy Centers with a major expansion planned over the next decade. Along with providing core services (e.g., case management, developmental assessments, parenting support), Judy Center partners work with and make referrals to local external agencies*.*

### Study design

This is a Hybrid Type 1 Implementation-Effectiveness trial designed as a two-arm randomized controlled trial (RCT), with individuals randomized (using REDCap-generated random numbers) via 1:1 allocation ratio to MB-VG or usual Judy Center services followed by delayed intervention receipt (receipt after data collection at 6-month time point). We selected an RCT design given our primary study aim to examine effectiveness of the MB-VG intervention. Women randomized to the usual care arm will be offered participation in MB-VG after completing their final data collection activity at 6 months post-randomization. We recognize this may result in MB-VG being offered beyond the period where there is greatest prevalence of depression among new mothers; however, this approach was endorsed by Judy Center partners. Specifically, they felt that in its absence some women may not consent to participation knowing they may not be able to receive MB-VG.

### Participants

Inclusion criteria for *perinatal intervention participants* include (1) 16 years of age or greater; (2) Identify as Latina; (3) Speaks Spanish/prefers to receive services in Spanish; (4) Pregnant or have a child 9 months or younger; At risk for a major depressive episode based on the following: mild to moderate depressive symptoms [5–14] on the Edinburgh Postnatal Depression Scale [52] and/or presence of risk factors for Major Depressive Episode (MDE) onset as measured by the Postpartum Depression Predictors Index-Revised (PDPI-R) [53–55]; ≥ 3 on PDPI-R when administered prenatally or ≥ 4 on the PDPI-R postnatally, or history of depression as indicated on the PDPI-R, regardless of score). Study exclusion criteria include (1) Individuals who score > 14 on the EPDS (likely meet criteria for a current major depressive episode for whom treatment would be recommended (vs. preventive intervention for depression); (2) Individuals who are not at risk for PPD—i.e., scores < 5 on the EPDS and under the aforementioned cutoffs on the PDPI-R and no personal history of depression; (3) Staff concern about cognitive limitations of a potential participant such that there is concern that they would not understand the intervention material; (4) Staff concern about a potential participant’s capacity to engage in a group intervention (e.g., concern about disruptive behavior); and (5) Unable to speak and understand Spanish.

As part of exploratory analyses, we will recruit *children of perinatal study participants* who are between the ages of 2.5 and 4.5 years at baseline. Inclusion criteria for intervention facilitators/staff and Judy Center directors include that the staff is a facilitator or co-facilitator of MB-VG groups or a director at a site participating in the study (either referring participants or delivering via their site). We plan to enroll a total of 486 study participants: (1) 300 perinatal individuals, (2) 150 children of perinatal individuals, (3) 24 MB-VG facilitators, and (4) 12 Judy Center directors.

### Recruitment and consent

**Fig. 1:**
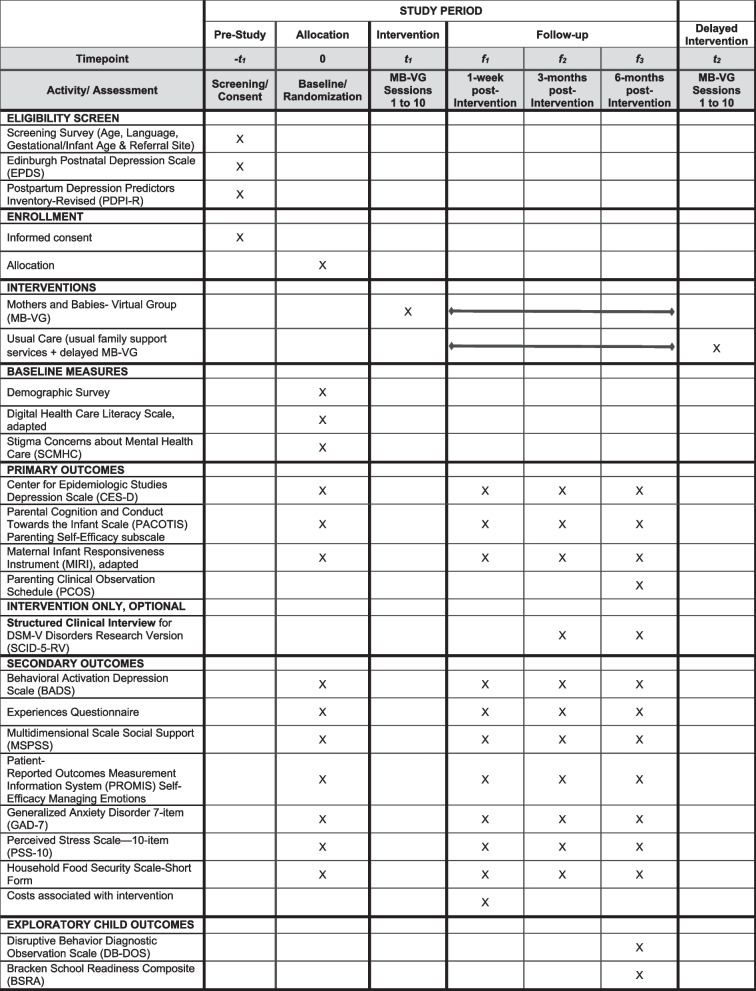
Schedule of screening, enrollment, interventions, and assessments

Recruitment will take place at participating Maryland Judy Centers. Planned recruitment strategies will include (a) flyers posted at Judy Center sites and (b) referrals from Judy Center staff. We anticipate most perinatal individuals will be recruited via referrals from Judy Center staff. The study team will work with each site to review eligibility criteria prior to recruitment. For women who are referred by staff and express interest, research staff will reach out by phone to discuss study procedures and administer a telephone screening, including the EPDS and PDPI-R. Individuals scoring 5–14 on the EPDS and/or ≥ 3/4 on the PDPI-R will be eligible for the study. Eligible participants will be able to consent either over the phone/zoom via oral consent, or independently through a unique electronic link sent by Research Electronic Data Capture (REDCap) [56], with research staff notifying participants as to their study arm assignment. Internet hotspots will be available for participants who would need them, and we budgeted a data plan stipend for all study participants, to offset costs related to study participation Fig. 1.

We anticipate that approximately 24 Judy Center staff will serve as MB-VG facilitators during this study. Each Judy Center staff member who facilitates a MB-VG cohort will be asked to provide informed consent prior to the first group they facilitate. A REDCap link with the informed consent form will be sent to and e-signed by the Judy Center staff/facilitator prior to facilitation. We will recruit the director from each of the 12 participating Judy Centers. Each director will be asked to provide informed consent prior to the completion of the baseline survey and prior to MB-VG implementation at their agency. The informed consent will be sent electronically via a REDCap link.

### Study intervention

#### MB-VG

Women randomized to the MB-VG arm will begin to receive the 10-session MB-VG intervention within 1 month of enrollment. These sessions are delivered weekly over a maximum of 15 weeks, with each session lasting between 60 and 75 min. All MB-VG groups will be delivered in Spanish by a trained MB-VG facilitator. Prior to each session, a member of the research team will test Zoom connections with each participant. Each session consists of cognitive-behavioral therapy-based content and is comprised of didactic and interactive content and ends with a “personal project” that encourages clients to practice using the core skills they learned in the session (see Table 1 below for core content found in each MB-VG session). In addition, a pediatrician will provide content related to nutrition, parenting, and child development. Text messages will be used as an implementation strategy to promote session attendance, encourage skill practice, and reinforce pediatrician content. Two bilingual Judy Center staff will co-facilitate each MB-VG intervention cohort. Based on previous MB research, attendance at over 50% of intervention sessions is defined as “full-dosage”; therefore, for this study, receipt of ≥ 6 sessions will constitute full-dose of MB-VG. We will document attendance at intervention sessions by asking one of the intervention co-facilitators to complete an attendance log for each MB-VG session. A member of the research team will also use Zoom login information to confirm the information found in the attendance logs. Participants who do not attend a session will receive a brief link via text to a video that reviews the session topics and key points. All videos for missed session are approximately 5 min or less.

#### Usual care

Women randomized to the usual family support services/usual care arm will receive Judy Center services without any MB-VG intervention during the study period (but will receive MB-VG after 6 months via our delayed intervention design, if interested). Judy Centers provide an array of services including parenting education, case management, family health and developmental assessments and referrals, and partnerships with childcare and education providers (e.g., Head Start).

### Training and supervision of MB-VG facilitators

All participating Judy Centers have been on MB-VG, or will be trained prior to beginning facilitation. Trainings provide six contact hours on:

1. Conceptual underpinnings of MB-VG (e.g., cognitive-behavioral therapy, psychoeducation, and attachment theories)
2. Format of the MB-VG instructor manual; specifically, for each new concept the manual presents: (a) key points, (b) rationale for concept, (c) considerations for facilitators, including sensitive issues that may emerge, and (d) a step-by-step script for introducing material.
3. Facilitation of virtual groups. For example, strategies for encouraging participation of more introverted group members and troubleshooting common technology issues.
4. Review of core CBT and attachment content in each MB-VG session. Each session is discussed from start to finish and various techniques are used to review MB-VG content (e.g., role plays, small group activities, structured discussion questions) to allow for multiple learning styles.
5. Importance of addressing food insecurity and parenting/child development and details on how the pediatrician is integrated into MB-VG delivery and procedures for referral to assistance with nutrition assistance program enrollment.
6. Overview of implementation protocol which includes information about (1) recruitment and referral processes, (2) preparing for virtual groups, (3) MB-VG session implementation (during and post sessions), (4) connecting participants to a resource advocate (FindHelp), (5) using HealthySMS for text messages, and (6) technology considerations.

Prior to delivering their first MB-VG cohort, staff who were initially trained > 6 months before study start will receive a 2-h refresher and protocol training via Zoom. This training will debrief facilitators’ prior experiences, if any, delivering MB-VG and discuss the study protocol and implementation logistics. Consistent with prior MB trials, while delivering their first MB-VG cohort, a facilitator will receive weekly supervision by research team members with expertise in the intervention. Within 1–2 weeks before implementation of the first group session the facilitator and co-facilitator(s) attend a 45–60-min virtual training to review the implementation protocol and technology considerations and needs.

Centers participating in the study that do not have bilingual staff (or do not have sufficient bilingual staff) will be able to refer participants to sites with trained bilingual staff, a key benefit of the virtual modality. To enhance the pragmatic nature of the study, staff with varied disciplinary backgrounds (e.g., child development, social work) will all be eligible to facilitate MB-VG virtual groups.

### Measures: Perinatal Latinas

Related to our effectiveness aims, we will collect data from perinatal individuals and their children. Each data collection procedure is listed below, and we will provide additional details on measures and time points in Table 2 below and in the SPIRIT flow diagram. Implementation outcome assessments are described in Table 3 below and guided by the Reach, Effectiveness, Adoption, Implementation, and Maintenance (RE-AIM) framework [48].

**Table 2:** Overview of measures and methods to assess intervention effectiveness with perinatal individuals and their children

Effectiveness outcome indicator	Measure(s)	Maternal self-report surveys	Observations	Structured clinical interview
**Baseline Measures**				
Demographic Survey	Developed by investigators	X		
Comfort with Technology	Adapted from Digital Health Care Literacy Scale [57]	X		
**Primary Outcomes**				
Depressive Symptoms	Center for Epidemiologic Studies Depression Scale (CES-D) [58]	X^**1**^		
Depressive Episodes	Structured Clinical Interview for DSM-V- Disorders Research Version (SCID-5-RV) [59]			X^**3**^
Parenting Self-Efficacy & Responsiveness	Parental Cognition and Conduct Towards the Infant Scale (PACOTIS) Parenting Self-Efficacy subscale [60]	X^**3**^		
Parenting Responsiveness	Maternal Infant Responsiveness Instrument (MIRI) [61]; Parenting Clinical Observation Schedule (PCOS) [62]	X^**3**^	X^**2**^	
**Exploratory Child Outcomes**				
Dysregulation	Disruptive Behavior Diagnostic Observation Scale (DB-DOS) [63, 64]		X^**2**^	
School readiness	Bracken School Readiness Composite (BSRC) [65, 66]		X^**2**^	
**Secondary Outcomes and Intervention Mechanisms**				
Behavioral activation	Behavioral Activation Depression Scale (BADS) [67, 68]	X^**1**^		
Decentering	Experiences Questionnaire [69]	X^**1**^		
Social support	Multidimensional Scale of Perceived Social Support (MSPSS) [70]	X^**1**^		
Mood management	Patient-Reported Outcomes Measurement Information System (PROMIS) Self-Efficacy Managing Emotions [71]	X^**1**^		
Anxiety Symptoms	Generalized Anxiety Disorder 7-item (GAD-7) [72]	X^**1**^		
Perceived Stress	Perceived Stress Scale—10-item (PSS-10) [73, 74]	X^**1**^		
Food Insecurity	Household Food Security Scale-Short Form [75]	X^**1**^		
Mental Health Treatment Stigma	Stigma Concerns about Mental Health Care [76]	X^1^		

^1^ Data collected at baseline, 1-wk. Post-intervention., 3 mos. Post-intervention., 6 mos. Post-intervention; ^2^ Data collected at 6 mos. Post-intervention; ^3^ Data collected at 3- and 6-mos. Post-intervention

**Table 3:** Overview of measures and methods to assess implementation outcomes

Implementation outcome indicator (Aim 2)	Measure(s)/metrics	Survey timing (respondents)	Sample explanatory/interview questions (Aim 3)	Interview timing (respondents)
**Reach**	% meeting eligibility criteria who enroll	Administrative/Ongoing	What explains barriers to enrollment?	Post-cohort implementation (facilitators) Post-completion of all intervention cohorts at early learning center (directors)
**Effectiveness**	See Table 2		What explains variability in effectiveness?	Post-completion of all intervention cohorts at early learning center (facilitators and directors)
**Adoption**	% sites & facilitators who begin MB-VG	Administrative/Ongoing	Barriers to center & facilitator adoption of MB-VG?	Post-completion of all intervention cohorts at early learning center (directors)
**Implementation**				
Implementation Readiness	Organizational readiness for implementing change [77]	Baseline (facilitators and directors)		
Acceptability	Acceptability of Intervention Measure (AIM) [78, 79] Attendance	Baseline (facilitators and directors) Post-cohort implementation (clients and facilitators) Post-completion of all intervention cohorts at early learning center (facilitators and directors)	Participant, facilitator, community, & site characteristics that explain variability in acceptability, feasibility, appropriateness? Rationale for MB-VG adaptations?	Post-cohort implementation (clients and facilitators) Post-completion of all intervention cohorts at early learning center (facilitators and directors)
Feasibility	Feasibility of Intervention Measure (FIM) [79] Attendance			
Appropriateness	Intervention Appropriateness Measure (IAM) [79]			
Fidelity/ Adaptation	Revised Cognitive Therapy Rating Scale (CTS-R) [80] Modified Adaptation Checklist (MAC) [81, 82]	Post-cohort implementation (facilitators) Post-completion of all intervention cohorts at early learning center (facilitators)		
Cost	Activity-Based Costing [83]	Baseline (facilitators and directors) Post-cohort implementation (clients, facilitators)	How did cost affect MB-VG engagement?	
Maintenance	Sustainment Measurement System Scale (SMSS) [84]	Post-completion of all intervention cohorts at early learning center (directors)	Barriers to maintenance?	

Legend. *C *Client, *F* Facilitator, *D* Director of Judy Center

### Self-report surveys

All participants will be asked to complete surveys at four time points: baseline (pre-intervention), 1-week post-intervention, 3 months post-intervention, and 6 months post-intervention to assess primary and secondary outcomes, as well as acceptability and feasibility. Data will be collected online and managed via REDCap. To minimize potential literacy issues, we will use REDCap’s text-to-speech function to allow participants to listen to audio survey questions if they prefer. For participants who strongly prefer to have a research team member administer surveys, a bilingual research assistant will conduct surveys by phone. We anticipate each survey will take 40–60 min, with participants remunerated $25–40 per survey, depending on the time point.

### Structured clinical interviews

To assess onset of major depression, all participants will be asked to complete the Mood Disorders module of the Structured Clinical Interview for DSM-V Diagnosis, Research version (SCID-5-RV) [85], a semi-structured interview for DSM-V Axis I diagnoses. The SCID will be administered at the 3- and 6-month post-intervention time points. Participants will be compensated $25 for completing the clinical interview at 3-month, and $30 for completing a clinical interview at 6-month post-intervention.

### Virtual observations

Related to our exploratory aim, participants with a child aged 2 ½–4 ½ will be invited to complete a virtual Zoom observation at the 6-month post-intervention time point after they complete their SCID. Observations will be video recorded to facilitate coding, with coded data entered into REDCap for analysis. Based on 2020 Judy Center data, we anticipate ~ 50% of families (*n* = 150) will have a child aged 2 ½–4 ½, who will provide data for this exploratory aim. Participants who complete virtual observations will be compensated $20.

### Qualitative interviews

We will recruit a stratified purposive sample (i.e., sampling from each participating Judy Center, selecting for variability in implementation and effectiveness outcomes) of up to 60 perinatal intervention participants for a semi-structured interview to further explain outcomes (e.g., variability in effectiveness or satisfaction), stopping if we reach data saturation (e.g., no significant new data after several interviews) earlier. Participants who complete qualitative interviews will be compensated $25.

### Measures: MB-VG Facilitators and Center Directors

All MB-VG facilitators (anticipated *n* = 24) will complete a baseline self-report survey through REDCap which includes demographic and pre-implementation questions assessing readiness, feasibility, acceptability, and appropriateness of the intervention as well as anticipated barriers. Additionally, each facilitator will be asked to complete a self-report survey after each MB-VG cohort they facilitate assessing implementation outcomes assessing feasibility, acceptability, and appropriateness of the intervention as well as implementation barriers, with a final implementation survey within 1 month after once all study cohorts are completed at that site. All MB-VG facilitators (*n* = 24) will complete a semi-structured interview after they complete their first MB-VG cohort and within 1 month after MB-VG study implementation is completed at that site.

### Fidelity assessment of intervention groups

All MB-VG sessions will take place via Zoom, with audio of each session transcribed in Spanish using Zoom’s transcription setting, and then uploaded to a secure online folder accessible only to research staff. Each session will be reviewed to document participant attendance and session length, with a random sample of 20% reviewed in depth to assess fidelity. Fidelity will be assessed using the Revised Cognitive Therapy Rating Scale (CTS-R) [80], previously modified to align with MB. Independent raters will assess fidelity by reviewing audio recordings of intervention sessions and documenting on a 3-point scale the extent to which each topic in a MB-VG session was covered. Adaptations will be assessed by asking facilitators to complete the Modified Adaptation Checklist (MAC) [81, 82] after each cohort they lead; the MAC will assess adaptation in 14 different ways (e.g., skipped topics, modified activities) and for each adaptation reported, whether the adaptation was planned. Adaptations will be explored in semi-structured interviews with facilitators and surveys, guided by Framework for Modification and Adaptations [86],

All Judy Center directors (*n* = 12) will be asked to complete a baseline REDCap survey to assess potential barriers to MB-VG implementation and includes demographic and additional pre-implementation questions assessing readiness, feasibility, acceptability, and appropriateness of the intervention as well as anticipated barriers, as well as a final implementation survey within 1 month after all study cohorts at that site are completed. The final survey will assess director perception of feasibility, acceptability, appropriateness of the intervention, and implementation barriers as well as anticipated barriers to sustainment. All Judy Center directors (*n* = 12) will also complete a semi-structured interview after MB-VG implementation is completed.

### Blinding

Because of the nature of the intervention, we will not be able to blind study participants, facilitators, and RAs assisting with the intervention. However, research staff who will be administering the Center for Epidemiologic Studies Depression Scale (CES-D), Parental Cognition and Conduct Towards the Infant Scale (PACOTIS) Parenting Self-Efficacy subscale, Maternal Infant Responsiveness Instrument (MIRI), SCID assessment, Disruptive Behavior Diagnostic Observation Scale and Bracken Basic Concept Scale School Readiness Composite will be blinded to study condition. Research staff who will be coding the DB-DOS and Bracken Basic Concept Scale School Readiness Component will also be blinded to study condition.

### Statistical analysis

Power calculations were conducted focusing on the effectiveness aim using maternal self-report of depressive symptoms (measured by the Center for Epidemiologic Studies-Depression scale (CES-D)) and parenting practices (measured by the Parental Cognition and Conduct Towards the Infant Scale (PACOTIS)), assuming power = 80%, a two-tailed test with an alpha of 0.05. To do these power calculations, we used the PowerUp tool. We plan to recruit 300 participants for the trial, 150 participants per condition. Based on these numbers, we expect a minimal detectable effect size of 0.241. Because of the longitudinal nature of the study, we expect some attrition (~ 20%). Accounting for attrition, we can expect a minimal detectable effect size of 0.269. Based on prior research, we expect small to moderate effect sizes, which we will have the ability to detect. We believe clustering by group will be very small and thus do not take it into account in the power analysis. Doing so would lead to a small increase in the minimal detectable effect sizes (e.g., increasing from 0.241 to 0.284).

### Missing data

Sample size and power calculations were completed under the assumption of 20% attrition for primary outcome analyses. Thus, while we anticipate missing data, we still expect adequate power to detect differences between study arms. We will examine rates of missing data for all variables and determine whether rates vary by participant characteristics, Judy Center program, or study arm. These analyses will indicate the extent to which missing data could bias results. We will attempt to collect data at each time point regardless of participants’ engagement in earlier data collection efforts. To minimize missing data due to loss to follow-up, we will devote considerable attention to promoting study retention. The planned mixed effects models are generally robust for unbalanced data across study time points. However, we plan to apply multiple imputation to test for bias introduced by missing data in sensitivity analyses; we will impute at least five datasets to generate an estimated average intervention effect.

We plan to perform all analyses using both the intent-to-treat (ITT) approach and a dose–response approach (treating dosage as a continuous variable of 0–10 sessions attended), adjusted for propensity scores. The dose–response approach will examine the relationship between number of MB-VG sessions attended (dosage) and outcomes, using propensity scores to match intervention participants to control-group participants with a similar propensity to take up a given level of intervention. This will reduce the influence of self-selection bias in intervention take up on the estimates of dosage effects. We plan to stratify analyses by whether participants are prenatal vs postpartum and whether participants are primiparous or multiparous.

Interim analysis will be performed to ensure safety. This analysis will focus on whether participating in the intervention has any significant iatrogenic effects. Individuals who score > 40 on the CES-D at any assessment timepoint will be flagged, since this level of symptomatology can be considered “very severe”; these participants will be contacted by the research team per procedures described below in the " Safety considerations and procedures" section. The safety analysis dataset will include those participants who had at least one session of the study intervention. No other formal planned interim analyses are planned.

### Quantitative analysis

Descriptive statistics will summarize baseline characteristics overall and by study arm (i.e., usual family support services vs. MB-VG). As appropriate, mean ± standard deviation (or median [inner quartile range]) will be used in cases of skewed or non-normal empirical distributions and frequency (proportions) will summarize continuous and categorical data, respectively. Analyses will employ normal theory methodology as appropriate, and in cases of violations of assumptions, transformations, and/or nonparametric analyses may be utilized. Analyses will proceed at the participant level, and all hypothesis tests will assume a two-sided 5% level of significance. We will apply the Benjamini–Hochberg correction to control family-wise type I error rate to 5%. We plan to perform all analyses using both the intent-to-treat (ITT) approach and a dose–response approach, adjusted for propensity scores. Multilevel analysis, i.e., generalized mixed effects models, will estimate baseline and multivariate adjusted between-group differences, adjusted odds ratios (AORs), and 95% confidence intervals (CIs) using individual-level data but considering the hierarchical and clustered structure of data, with participants clustered by group (i.e., Judy Center). We believe clustering by group will be very small and thus do not take it into account in the power analysis. Doing so would lead to a small increase in the minimal detectable effect sizes (e.g., increasing from 0.241 to 0.284). Continuous outcomes will use a model that assumes normally distributed outcomes, and appropriate transformations of the data will be made to improve normality. Binary outcomes will be assessed using a logistic regression-based multilevel model.

*Effectiveness outcomes*: Aim 1 will be tested by examining the coefficient of the treatment vs. control indicator in the random effects model. We will include random intercepts for group and instructors and will also investigate whether a random slope for group is appropriate for the intervention effect. Intervention group, covariates measured at pretest (especially the pretest measures of the outcome, as available), the Judy Center the participant was recruited from, and instructor characteristics will be included as fixed effects. Likelihood ratio tests (significance criterion, *p* < 0.05) will be used to simplify the models following a backward selection strategy. Although the primary analyses will consider each set of follow-up outcomes separately, controlling for pre-test measures in each analysis, we will also consider the use of longitudinal growth models to model the growth in knowledge over the three time points. Primary endpoint #1 is depressive symptoms, as measured by the Center for Epidemiologic Studies-Depression scale (CES-D). A total continuous score (0–60) is calculated for the CES-D. Primary endpoint #2 is depressive episodes as measured by the SCID. The SCID is used to assess the presence/absence of major depression, making it a binary outcome (yes/no). Primary endpoints #3 and #4 are parenting self-efficacy measured via the PACOTIS and parenting responsiveness measured via the MIRI and PCOS (used as a separate coding system when coding the DB-DOS observation assessment). A mean score is calculated for each of the PACOTIS and MIRI subscales. Secondary endpoint #1 is anxiety symptoms, measured via the GAD-7. A total continuous score is calculated for the GAD-7. Secondary endpoint #2 is perceived stress, measured via the PSS-10. A total continuous score is calculated for the PSS-10.

Covariates will be finalized in a Statistical Analysis Plan but based on our prior studies [80, 87] will include (1) whether participant is a first-time mother (e.g., current number of children = 0 at baseline; (2) education (at least some high school vs less than high school); (3) mental health service utilization (we will control for enrollment with a therapist for counseling at baseline and medication for depression at baseline).

Outliers will be assessed to determine whether it is a data entry error or some other protocol error. If not, sensitivity analyses will be conducted to assess the impact of the outliers. Because this is an intent-to-treat analysis, non-adherence will not be formally dealt with. Reasons for loss to follow-up will be explored and we will determine if any key factors contributed to dropout or attrition. Results of initial analyses will determine the best method to handle missing data in the main study analyses.

Analysis of the secondary endpoints will be examined in a manner like those described above related to analysis of our primary endpoints. Primary and secondary outcomes will be assessed utilizing mixed effects logistic regression (when outcomes are binary) and mixed effects linear regression (when outcomes are continuous). Subgroup analysis will follow the same analytic protocol, beginning with the addition of an interaction term between intervention status and the subgroup variable. This will be followed by multiple group analysis.

For *Implementation Outcomes*, mean scores of Acceptability of Intervention Measure, Implementation Appropriateness Measure, and Feasibility of Intervention Measure will be calculated for each perinatal participant and facilitator participant to assess MB-VG *acceptability, appropriateness*, and* feasibility*, respectively. We will use descriptive statistics to examine variability in implementation outcomes by respondent type and participant characteristics. We will examine variability in session attendance and utilization of Resource Advocate by participant characteristics. *Fidelity* will be calculated via a mean adherence and competency rating for each rated session with linear mixed models used to evaluate site, participant, and facilitator characteristics’ influence on these outcomes. *Adaptation* will be calculated using descriptive statistics to assess frequency of adaptations with latent class analysis examining whether adaptations group together in underlying patterns across facilitators or Judy Centers. *Cost* implementing MB-VG will be generated by deriving a per-hour salary estimate for each facilitator, Resource Advocate, and pediatrician. When possible, we will obtain actual salaries (see baseline survey for facilitator participants and for director participants); if these salaries are unavailable, we will use state-specific salary estimates based on position title and degree from the Bureau of Labor Statistics for the relevant year. We then will multiply wage rates plus fringe benefits by the time spent engaged in MB training and delivery. Once total costs are calculated, we will calculate a per-organization and per-participant cost of MB-VG. We will aggregate labor and non-labor cost data to measure the cost of implementation from a societal perspective inclusive of organization and participants, as well as reported separately by group. *Maintenance* will be calculated by generating mean scores for the overall SMSS and each of its domains.

*Exploratory outcomes*: We will conduct analyses related to our exploratory objective assessing child dysregulation and school readiness among children aged 2.5–4.5 years. For the Disruptive Behavior Diagnostic Observation Scale (DB-DOS), we will conduct a series of ANOVAs examining differences between intervention and control children on each DB-DOS subscale: anger modulation, behavioral regulation, and competence. For the Bracken School Readiness Composite, we will conduct an ANOVA comparing intervention and control children on their overall school readiness composite score.

### Qualitative analysis plan and mixed methods integration

Audio files will be transcribed (and translated, when applicable). Data will be organized with NVivo Version 12 and analysis guided by the process of constant comparison [88]. Transcript coding will be primarily deductive based on implementation domains (Reach, Adoption, Implementation, Maintenance) and constructs from the Consolidated Framework for Implementation Research constructs [89] but we will use inductive coding when these codes do not appear to adequately explain the aspect of implementation described. Two team members will independently code each transcript with regular meetings used to discuss updates to the codebook and gain consensus on any disagreements in coding. Themes will be developed by reviewing excerpts associated with codes, visually mapping relations between codes, identifying broader domains suggested by codes and discussing how codes do or do not relate to constructs of interest. We will merge quantitative and qualitative data using joint displays demonstrating key quantitative and qualitative findings [89] and linking contextual factors to understand variability in effectiveness and implementation outcomes. Joint displays facilitate assessment of convergence or divergence in data within constructs and can elucidate necessary next steps to build upon study results [50].

### Safety considerations and procedures

Any participant whose responses on the EPDS (or subsequent assessments) indicate that they have had thoughts about their death or suicide within the past month will be asked additional questions to determine the suicidality degree, including whether they have experienced thoughts of their own death, thoughts about suicide, a specific plan or details about how they might take action, and any attempts they have made to hurt themselves. If assessments have been self-administered (e.g., EPDS), participants will via REDCap automatically receive information about 24/7 Spanish-language resources (e.g., National Suicide Prevention Hotline, Postpartum Support International, and the National Maternal Mental Health Hotline). Participants will be required to acknowledge receipt of these resources. In addition, designated study personnel will be immediately alerted by email and text via the survey notification function in REDCap and will reach out to and assess participants within 24 h. In this assessment, participants will be asked additional questions to determine the suicidality degree, including whether they have experienced thoughts of their own death, thoughts about suicide, a specific plan, or details about how they might take action, and any attempts they have made to hurt themselves. Study staff will also reach out to the referring Judy Center to work collaboratively to coordinate referrals for additional services if needed (case management is a core component of the Judy Center). In addition to events prompting safety protocol as above (reporting suicidal ideation on assessments), individuals who have a clinically significant finding of a CES-D score > 40 at any time point will trigger the use of our safety protocol (including text alert to PI via REDCap if completed independently) to ascertain whether there is any risk for self-harm. We will do analysis quarterly to assess for any iatrogenic effects. Text messages in MB-VG are designed to be unidirectional and primarily focused on reminding participants to practice skills and provide reminders about sessions and resources. However, it is possible that participants may respond to messages. Participants will be notified that the system is automated and that it does not constitute direct communication with a provider or therapist. Additionally, the platform will be programmed such that and research staff will be notified via text message and/or email if a keyword is texted via the SMS platform that express potential for self-harm, such as “die,” “suicide,” and “kill”. Additionally, if the participant types one of these keywords, a text message will be automatically sent to the participant like those described above. Prior to initiating the study/intervention, the research team will work with each Judy Center to confirm their client risk assessment procedures, which typically include immediate notification of relevant center staff (e.g., case manager or center director/supervisor), risk assessment, provision of local resources, or emergency referral.

*Criteria for discontinuation from intervention*: Participants will be discontinued from the intervention if they (1) develop a severe medical (e.g., bedrest due to pregnancy complications) or mental health condition (e.g., psychosis, active mania) preventing the participant from engaging in the intervention and/or understanding the intervention; (2) indicate a preference not to continue in the intervention; or (3) experience pregnancy loss or neonatal/infant loss. In the case of a participant experiencing a pregnancy or infant loss, the study team will discuss intervention continuation with the participant. We anticipate most participants will prefer not to continue in the intervention given its focus on mothers and babies. When a subject discontinues from MB-VG but not from the study, remaining study procedures will be completed as indicated by the study protocol. The exception will be the removal of all parenting-related assessments for participants who experienced a pregnancy or infant loss.

*Adverse event monitoring*: For this trial, there are three potential adverse events that will be monitored and logged: (1) movement into the severe depressive symptom range, (2) suicidal ideation, and (3) potential child abuse/neglect. There is nothing about the MB-VG intervention (including prior related MB studies) that suggest that they will generate any of these adverse events, but each will be monitored nonetheless. All adverse events occurring while on study will be documented in REDCap appropriately regardless of relationship. We will follow Institutional Review Board Adverse Event Reporting guidelines and will also report adverse events to the study Data and Safety Monitoring Board.

## Discussion

To our knowledge, this study will be one of the first to test the efficacy of a group-based virtual *perinatal depression* intervention to Latina immigrants, for whom stark *disparities* exist in access to *mental health services* [10]. Use of a virtual format for mental health interventions, including group interventions, expanded rapidly during the COVID-19 pandemic [40, 90]. In the virtual format will facilitate the incorporation of supplementary content (e.g., group discussions with a pediatrician, inclusion of a resource advocate) that may be of particular benefit to immigrant Latinas. Our hybrid effectiveness-implementation design will allow rigorous examination of barriers and facilitators to delivery of the intervention package (including supplemental components) which will provide valuable information on factors influencing intervention effectiveness and the scalability of intervention components. Similarly, implementation of the intervention in early childhood programs provides a unique setting in which to reach individuals who might not otherwise access mental health services. Addressing maternal depression has been identified as a critically needed enhancement to early childhood programs [91] yet most previous RCTs have focused on home-based programs. [92].

There are several potential challenges and practical issues to consider. First, we expect there will be some degree staff turnover at Judy Centers, turnover is an important and prevalent challenge to scaling and sustaining evidence-based mental health interventions [93]. Given this, we plan to explicitly assess center director perceived barriers to sustainment of the intervention. Challenges recruiting perinatal participants may occur which could lead to lag time between study enrollment and intervention delivery. To minimize attrition and decrease wait time between enrollment and intervention start, we will allow for smaller (e.g., 4–5 participants) intervention cohorts and for cohorts combining individuals from across Judy Centers. The virtual format facilitates combining individuals from across Judy Centers; with the impact of blended versus single-center cohorts on effectiveness and implementation explored through our qualitative data collection activities. Additionally, as multiple Judy Center staff will be trained on MB-VG, it is possible they will use intervention language and activities during their work with participants randomized to the usual care arm. Study investigators have successfully addressed this in previous trials by clearly communicating study protocols to all agency staff. Moreover, our delayed intervention design means that control participants will be able to receive MB-VG thus minimizing concern among staff that participants will not benefit from intervention content.

### Data management

Study participant research data, which is for purposes of statistical analysis and scientific reporting, will be transmitted to and stored at Johns Hopkins University. This will not include the participant’s contact or identifying information. Rather, individual participants and their research data will be identified by a unique study identification number. The study data entry and study management systems (REDCap for quantitative data) used by Johns Hopkins University research staff will be secured and password protected. At the end of the study, all study databases will be de-identified and archived.

### Data safety and trial monitoring

An independent Data Safety Monitoring Board (DSMB) has been formed and will meet once per year to monitor adverse events and trial progress. The three-person DSMB is Lauren Osborne, MD (Chair); Rhonda Boyd, PhD; Mary Sammel, ScD. Protocol modifications will be reported to the DSMB and Johns Hopkins IRB, with any significant protocol changes related to participants’ trial involvement shared with already enrolled study participants.

### Additional trial oversight

There is not a formal Trial Steering Committee. The team organization is divided such that the Johns Hopkins University team oversees and collects data related to effectiveness of the intervention (e.g., collection of primary outcomes) and the Northwestern University team oversees and collects data related to intervention implementation (e.g., fidelity monitoring, facilitator and director self-report surveys and interviews, and qualitative interviews with intervention participants). The Johns Hopkins University research team is responsible for managing recruitment and consent of perinatal participants and has responsibility for overall oversight of data collection and management of quantitative study data. The Northwestern University team has responsibility for collection, management, oversight, and analysis of qualitative data, as well as coding data from observational assessments. The Northwestern and Johns Hopkins University Study teams meet weekly, and co-investigators meet monthly. In addition, separate weekly supervision is provided for the Johns Hopkins University research team members completing the SCID and the DB-DOS/Bracken.

### Stakeholder and public involvement group

The trial has two advisory boards, one comprised of Judy Center staff participating in the trial, and one (facilitated in Spanish) comprised of participants in the pilot study. Both boards meet quarterly for the duration of the study and provide input and recommendations to the study team on recommendations for recruitment, study challenges, and need for adaptation of measures.

### Status of the trial

Protocol version #6, with most recent approval February 1, 2024. Participant recruitment began in September 2023. It is expected that recruitment will continue through September 2025.

## Supplementary Information

Supplementary Material 1.

Supplementary Material 2.

## Data Availability

The datasets used and/or analyzed during the current study are available from the corresponding author on reasonable request. The investigators will develop a transportable de-identified database, codebook, and mechanism by which data can be shared with other investigators upon approval of the study’s research team (Co-Investigators) and Institutional Review Board. Permission to transmit de-identified data will be included in the informed consent.

## Abbreviations

AOR: Adjusted odds ratio
BSRC: Bracken School Readiness Composite
CBT: Cognitive behavioral therapy
ANOVA: Analysis of variance
CES-D: Center for Epidemiologic Studies- Depression Scale
CI: Confidence interval
DB-DOS: Disruptive Behavior Diagnostic Observation Scale
DSMB: Data Safety Monitoring Board
DSM-V: Diagnostic and Statistical Manual of Mental Disorders, Fifth Edition
EPDS: Edinburgh Postnatal Depression Scale
GAD-7: Generalized Anxiety Disorders-7
IRB: Institutional Review Board
ITT: Intention-to-treat
MB: Mothers and Babies
MB-VG: Mothers and Babies – Virtual Group
NCT: National Clinical Trial
NIH: National Institutes of Health
NIMHD: National Institute of Minority Health and Health Disparities
PA: Pleasant Activity
PACOTIS: Parental Cognitions and Conduct Towards the Infant Scale
PDPI-R: Postpartum Depression Predictors Inventory- Revised
PI: Principal Investigator
PPD: Postpartum depression
PSS-10: Perceived Stress Scale—10-item
RA: Research assistant
RCT: Randomized clinical trial
RE-AIM: Reach, Effectiveness, Adoption, Implementation, Maintenance Framework
SCID: Structured Clinical Interview for DSM-5
SNAP: Supplemental Nutrition Assistance Program
US: United States
