# Supplementary material for: Mothers and Babies Virtual Group (MBVG) for perinatal Latina women: study protocol for a hybrid type-1 effectiveness-implementation randomized controlled trial
Source: Trials. 2024 Sep 11;25:606. doi: 10.1186/s13063-024-08423-z (PMC11391742; doi:10.1186/s13063-024-08423-z)
Supplement: Supplementary file 2 — Supplementary Material 2. [file 13063_2024_8423_MOESM2_ESM.docx]

**Supplemental Table 1: Content of MB-VG Text Messages**

| **Msg Bank** | **Session** | **Session Topics** | **Skill Reinforcement** | **Homework Reminder** | **Mindfulness Content** | **Pediatrician** |
| --- | --- | --- | --- | --- | --- | --- |
| **Intro MB &**  **session 1 TXT** | **1** |  | Welcome to Mothers & Babies (MB) TXT! You are receiving this text messages because you are receiving services from a Judy Center and are signed up to receive messages. | You will receive 4 texts after each MB session with reminders, resources, and tips to add to your toolkit. | You can text STOP at any time to end the messages. |  |
|  | 1 | *Course Introduction*  Stressors that affect Mom-Baby Relationship  How can MB help  MB Overview  Mood & Personal Reality  Quick Mood Scale | Everybody has stress. It affects how you feel and can affect your baby. Do something today to manage stress, like taking a few deep breaths. | Remember to keep track of how you are feeling by rating your mood at the end or start of each day. | Here is a brief guided breathing meditation to introduce you to mindfulness practice.    [MB Breath Awareness](https://www.mothersandbabiesprogram.org/?attachment_id=1353) | Your facilitator can refer you to a resource advocate to help you connect to resources! You can learn more about the benefits of SNAP and how you can enroll by clicking on this link: [LINK](https://www.mothersandbabiesprogram.org/wp-content/uploads/2023/10/Pediatrician-Participant-Worksheet-1.6.pdf) |
| **TXT 2** | **2** | *Pleasant Activities*  *-*Quick Mood Scale Review  What we do affects how we feel  What do you like to do? PA | Doing one pleasant activity can lead to more. Pick one thing to do to de-stress. Pleasant activities can be low or no cost, brief, and part of  daily routines. | What do you enjoy doing by yourself, with others, and with your baby?  Think about it or write it down. | We reviewed a new mindfulness practice called the Body Scan, which also uses breath awareness.    [MB Body Scan](https://www.mothersandbabiesprogram.org/?attachment_id=1385) | It is important for babies to get good nutrition – not too little, not too much, just the right amount. Click this link to see hunger and satiety cues: [LINK](https://www.mothersandbabiesprogram.org/wp-content/uploads/2023/10/Pediatrician-Participant-Worksheet-2.5.pdf) |
| **TXT 3** | **3** | *Pleasant Activities*    *-*Review PA checklist    - Overcoming obstacles    - What do babies like to do?    - How do babies learn? | Babies learn by playing. Doing pleasant activities with you supports their development. Notice how babies like to play. | Remember to do one pleasant activity this week. Fit it into your week by planning when to do it. | The same way you practiced your breathing, and the sensations in the body, walking meditation is an opportunity to bring our awareness to all of our senses.    [MB Walking Meditation](https://www.mothersandbabiesprogram.org/?attachment_id=1431) | Babies and children learn by interacting with others – by listening to people, talking and playing. Click this link to learn more about how you can promote your child’s language development: L[INK](https://www.mothersandbabiesprogram.org/wp-content/uploads/2023/10/Pediatrician-Participant-Worksheet-3.pdf) |
| **TXT 4** | **4** | *Thoughts*  *-*What are thoughts?  - Helpful thoughts | People have helpful and unhelpful thoughts and both kinds of thoughts affect how we feel. Make an effort to notice your thoughts today. | What helpful or unhelpful thoughts have you had this week about being pregnant or becoming a mom? Write them down or think  about them. | Mindfulness activities such as “Leaves on a Stream” can help you begin seeing thoughts as words and images that come and go.    [MB Leaves on a Stream](https://www.mothersandbabiesprogram.org/?attachment_id=1459) | Remember crying is a baby’s form of communication to express their needs. Click here to explore some common reasons why babies cry and how you can respond to their needs:  [Link](https://www.mothersandbabiesprogram.org/wp-content/uploads/2023/10/Pediatrician-Participant-Worksheet-4.5.pdf) |
| **TXT 5** | **5** | *Thoughts*  *-Types of harmful thought patterns*  *- Ways to change harmful thought patterns* | Try using one of these strategies when you notice unhelpful thoughts: self- instruction, time projection, thought interruption, or worry  time. | Try to notice the helpful and unhelpful thoughts you have. Try using a new strategy to increase helpful thoughts and to let unhelpful thoughts go. | Try this mindfulness practice to help ground your senses ([link](https://www.uclahealth.org/marc/mpeg/03_Complete_Meditation_Instructions.mp3)). | Remember, babies younger than 12 months should be fed infant formulas or breast milk. Click on the link to explore the different types of milk: [Link](https://www.mothersandbabiesprogram.org/wp-content/uploads/2023/10/Pediatrician-Participant-Worksheet-5.4.pdf) |
| **TXT 6** | **6** | *Thoughts*  *-*Promoting your child's healthy thinking  - Goals for my future and my baby's future | Your thoughts can affect your future and your baby’s future. Thinking about what you want in the future can help you to take steps to achieve your goals. | Keep track of your mood, notice how many helpful/unhelpful thoughts you have. | As you try to visualize you and your baby's ideal future, try taking a moment with the Walking Meditation and ground into your senses and your surroundings.    [MB Walking Meditation](https://www.mothersandbabiesprogram.org/?attachment_id=1431) | Sometimes we are worried that a baby doesn’t weigh enough, We also worry when babies gain weight too quickly. Take a look at the different factors that influence healthy child growth. [LINK](https://www.mothersandbabiesprogram.org/wp-content/uploads/2023/10/Pediatrician-Participant-Worksheet-6.5.pdf) |
| **TXT 7** | **7** | *Contact with Others*  -Relationship between mood & contact with others | Being with others can affect how you feel. Many people feel sad or depressed when they don’t have positive contacts or have negative  contacts with others. | Rate how you are feeling and the number of positive and negative contacts you have each day. Try to do this every day of this week. | Some mindfulness activities can help us recognize how we are all connected. Try this mindfulness practice focusing on compassion.    [MB Loving Kindness](https://www.mothersandbabiesprogram.org/?attachment_id=1526) | Setting limits helps children build self-control, take responsibility for their actions, and learn between right and wrong. Click here to review some tips for setting limits. [LINK](https://www.mothersandbabiesprogram.org/wp-content/uploads/2023/10/Pediatrician-Participant-Worksheet-7.5.pdf) |
| **TXT 8** | **8** | *Contact with Others*  -The people in my life  - People in my life and the ways they support me | Support from others is important for everyone. Positive contacts help you when life is challenging. Different people can provide support for different  things. | Think about pleasant activities you can do to meet people who might become part of your support network. It helps to have more supportive people in your life. | Role changes can be stressful. Mindfulness can help you pay attention to how your role change makes you feel. Try to tap into self-compassion.  [MB Loving Kindness](https://www.mothersandbabiesprogram.org/?attachment_id=1526) | Children may really like juice because it tastes good. However, too much juice can contribute to some children’s health problems. Here are some recommendations for daily juice consumption: [LINK](https://www.mothersandbabiesprogram.org/wp-content/uploads/2023/10/Pediatrician-Participant-Worksheet-8.5.pdf) |
| **TXT 9** | **9** | *Contact with Others*  Communication styles and your mood  Getting your needs met  Course review | It's important to get your needs met. Positive, clear, and direct requests are effective ways to communicate. Practice being assertive. | This week try to ask for something from anyone - even your home visitor! What way of asking will help get your needs met - passive, aggressive, assertive? | Mindfulness practice takes a few minutes & brings calmness to your day. Try these guided meditations for everyday situations ([link](http://marc.ucla.edu/mindful-meditations)). | Look at all the benefits of being bilingual. It helps children stay connected with their family, culture, and community. [LINK](https://www.mothersandbabiesprogram.org/wp-content/uploads/2023/10/Pediatrician-Participant-Worksheet-9.3.pdf) |
| **TXT 10** | **10** | Course review | You can create a healthy reality for you and your baby with your activities, your thoughts, and your contact with other  people. | Remember to review your [workbook](https://www.mothersandbabiesprogram.org/resource-category/mb-manuals/) to use the skills you learned or to use other skills you have not tried yet. | Save the mindfulness links in these MBTXT messages and use them whenever you want and need to!  [MB Breath Awareness](https://www.mothersandbabiesprogram.org/?attachment_id=1353) | Remember to utilize your pediatrician as a resource. Don’t be afraid to advocate for the quality of care you and your child deserve! |
| **EXIT TXT** |  |  | Congratulations on finishing MB! |  |  |  |
